# *In vivo *imaging of zebrafish retinal cells using fluorescent coumarin derivatives

**DOI:** 10.1186/1471-2202-11-116

**Published:** 2010-09-15

**Authors:** Kohei Watanabe, Yuhei Nishimura, Takehiko Oka, Tsuyoshi Nomoto, Tetsuo Kon, Taichi Shintou, Minoru Hirano, Yasuhito Shimada, Noriko Umemoto, Junya Kuroyanagi, Zhipeng Wang, Zi Zhang, Norihiro Nishimura, Takeshi Miyazaki, Takeshi Imamura, Toshio Tanaka

**Affiliations:** 1 Department of Molecular and Cellular Pharmacology, Pharmacogenomics and Pharmacoinformatics, Mie University Graduate School of Medicine, Tsu, Mie 514-8507, Japan; 2 Mie University Medical Zebrafish Research Center, Tsu, Mie 514-8507, Japan; 3 Department of Medical Chemogenomics, Mie University Venture Business Laboratory, Tsu, Mie 514-8507, Japan; 4 Department of Bioinformatics, Mie University Life Science Research Center, Tsu, Mie 514-8507, Japan; 5 Department of Translational Medicine, Mie University Graduate School of Medicine, Tsu, Mie 514-8507, Japan; 6 Corporate R&D Headquarters, Canon Inc., Ohta-ku, Tokyo 146-8501, Japan

## Abstract

**Background:**

The zebrafish visual system is a good research model because the zebrafish retina is very similar to that of humans in terms of the morphologies and functions. Studies of the retina have been facilitated by improvements in imaging techniques. *In vitro *techniques such as immunohistochemistry and *in vivo *imaging using transgenic zebrafish have been proven useful for visualizing specific subtypes of retinal cells. In contrast, *in vivo *imaging using organic fluorescent molecules such as fluorescent sphingolipids allows non-invasive staining and visualization of retinal cells *en masse*. However, these fluorescent molecules also localize to the interstitial fluid and stain whole larvae.

**Results:**

We screened fluorescent coumarin derivatives that might preferentially stain neuronal cells including retinal cells. We identified four coumarin derivatives that could be used for *in vivo *imaging of zebrafish retinal cells. The retinas of living zebrafish could be stained by simply immersing larvae in water containing 1 μg/ml of a coumarin derivative for 30 min. By using confocal laser scanning microscopy, the lamination of the zebrafish retina was clearly visualized. Using these coumarin derivatives, we were able to assess the development of the zebrafish retina and the morphological abnormalities induced by genetic or chemical interventions. The coumarin derivatives were also suitable for counter-staining of transgenic zebrafish expressing fluorescent proteins in specific subtypes of retinal cells.

**Conclusions:**

The coumarin derivatives identified in this study can stain zebrafish retinal cells in a relatively short time and at low concentrations, making them suitable for *in vivo *imaging of the zebrafish retina. Therefore, they will be useful tools in genetic and chemical screenings using zebrafish to identify genes and chemicals that may have crucial functions in the retina.

## Background

The similarities in the morphologies and functions of the zebrafish and human retinas have made the zebrafish visual system a useful research model [1-4]. Like the human retina, the neuronal cell bodies are precisely organized in three major laminae, the ganglion cell layer (GCL), inner nuclear layer (INL) and outer nuclear layer (ONL) [1]. These three laminae are separated by plexiform layers, the inner plexiform layer (IPL) and outer plexiform layer (OPL), which mainly contain neuronal projections [1]. Furthermore, the zebrafish has a cone-dense retina and thus has rich color vision, providing an advantage over nocturnal rodent retina studies [2,3]. The organization of the genome and the genetic pathways controlling signal transduction and retinal development are also highly conserved between zebrafish and humans [4]. Because zebrafish are highly tractable to both genetic and chemical manipulation, many genetic and chemical screenings have been performed [1-7]. From these screenings, a number of genes and chemicals have been identified that could affect the structures and functions of the vertebrate retina [1-5].

Retinal research has been facilitated by improvements in imaging techniques. Multiple technical developments have permitted the visualization of retinal cell structures and their dynamics *in vitro*, *ex vivo *and *in vivo *[8]. *In vitro *approaches such as immunohistochemical analyses allow the labeling of certain retinal cell types [9]. DiOlistic labeling with fluorescent dyes is an *ex vivo *approach to display arbor morphologies of the retina by labeling single cells discretely [8]. These *in vitro *and *ex vivo *approaches can be used with multiple probes to detect changes in several retinal cell types simultaneously [9,10]. However, these approaches are labor-intensive and have relatively low throughputs. As an *in vivo *approach, stable transgenic zebrafish lines expressing fluorescent proteins such as green fluorescent protein (GFP) have been used [11,12]. In these transgenic lines, fluorescent proteins are expressed in specific cell types, such as rod photoreceptors [13], UV-sensitive cone photoreceptors [14], subtypes of bipolar cells [15] and retinal ganglion cells (RGC) [11,12]. Although these transgenic lines can be used for high-throughput *in vivo *screening by assessing the changes in the fluorescent signals in the retina [11,12], the assessments are usually restricted to the cells expressing the fluorescent proteins.

Another technique for *in vivo *imaging is vital staining of the zebrafish retina using fluorescent small molecules [16,17]. Zebrafish larvae absorb small molecules present in the surrounding water through their skin and gills [18]. Fluorescent sphingolipids such as Bodipy-ceramide have been used as labeling agents for *in vivo *imaging of the zebrafish retina [19,20]. Fluorescent sphingolipids are inserted into the plasma membrane of many cells in zebrafish, allowing the cellular and axon layers of the zebrafish retina to be visualized [20]. However, since fluorescent sphingolipids also localize to the interstitial fluid of zebrafish larvae, the whole larvae are stained *en masse *[16]. Therefore, *in vivo *imaging of the zebrafish retina using fluorescent sphingolipids requires staining for several hours and at high concentrations (50-100 μM) to achieve visualization.

In this study, we screened fluorescent coumarin derivatives that could resolve these problems. It has been shown that coumarin derivatives can reach the mammalian brain by passing through the blood-brain barrier (BBB) and have potential as therapeutic agents for autoimmune encephalomyelitis [21] and amyloid imaging agents for Alzheimer's disease [22]. Since the BBB and blood-retinal barrier (BRB) are both endothelial barriers where tight junctions between the endothelial cells seal the vascular lumen [23], we hypothesized that coumarin derivatives would also be delivered into the retina. This screening identified four coumarin derivatives that are suitable for *in vivo *imaging of the zebrafish retina.

## Results

### Identification of coumarin derivatives suitable for in vivo imaging of the zebrafish retina

**Figure 1:**
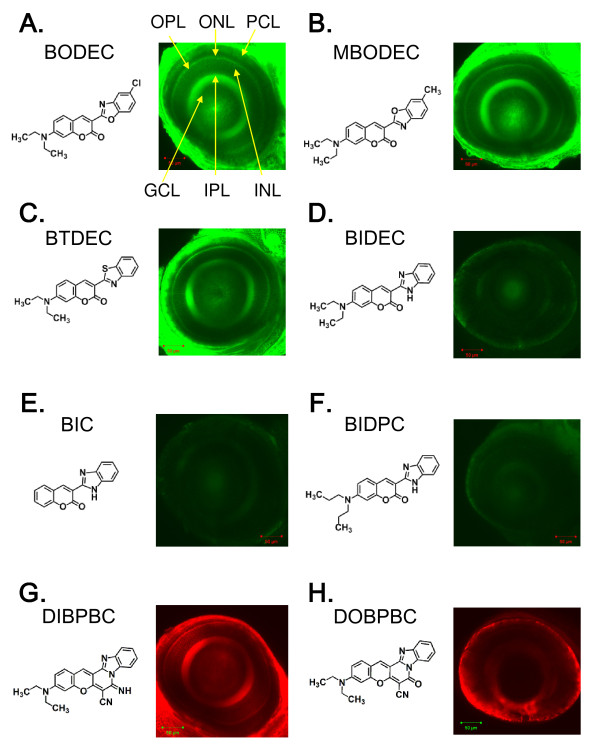
**Identification of coumarin derivatives visualizing the multiple layers of the zebrafish retina *in vivo***. Zebrafish larvae at 6 dpf were stained with BODEC (A), MBODEC (B), BTDEC (C), BIDEC (D), BIC (E), BIDPC (F), DIBPBC (G) and DOBPBC (H). The retinas were visualized by confocal laser scanning microscopy. The zebrafish retinas are clearly visualized by BODEC (A), MBODEC (B), BTDEC (C) and DIBPBC (G). OPL: outer plexiform layer; ONL: outer nuclear layer; PCL: photoreceptor layer; GCL: ganglion cell layer; IPL: inner plexiform layer; INL: inner nuclear layer.

To identify fluorescent dyes that could preferentially stain retinal cells of living zebrafish, we screened eight coumarin derivatives (Table 1) as described in Methods. As shown in Figure 1, 3-(2-benzoxazolyl)-7-(diethylamino)-coumarin (BODEC), 3-(6-methyl-2-benzoxazolyl)-7-(diethylamino)-coumarin (MBODEC), 3-(2-benzothiazolyl-7-(diethylamino)-coumarin (BTDEC) and 3-(diethylamino)-7-imino-7H-(1)benzopyrano(3',2':3,4)pyrido(1,2-a)benzimidazole-6-carbonitrile (DIBPBC) strongly stained multiple layers of the retina in living zebrafish. The IPL and OPL were visualized with strong fluorescence, whereas the fluorescence in the GCL, INL and ONL appeared reticulated. The IPL and OPL are synaptic layers that contain neuronal projections from the INL and GCL, and from the ONL and INL, respectively. The strong fluorescence in the IPL and OPL and reticular staining of the GCL, INL and ONL suggest that the coumarin derivatives may stain the plasma membranes of neuronal cells in the zebrafish retina. Consistent with this notion, the photoreceptor cell layer (PCL), which is the outer part of the ONL and contains many plasma membranes, was also visualized by coumarin derivatives with relatively strong fluorescence (Figure 1A-C and 1G). The fluorescent signals in the retinas stained with 3-(2-benzimidazolyl)-7-(dipropylamino)-coumarin (BIDEC), 3-(2-benzimidazolyl)-7-coumarin (BIC), 3-(2-benzimidazolyl)-7-(dipropylamino)-coumarin (BIDPC) and 3-(diethylamino)-7-oxo-7H-(1)benzopyrano(3',2':3,4)pyrido(1,2-a)benzimidazole-6-carbonitrile (DOBPBC) were comparatively weaker (Figure 1D-F and 1H).

**Table 1:** Coumarin derivatives used in this study

chemical name	abbreviation	MW	Ex	Em	FI
3-(2-Benzothiazolyl-7-(diethylamino)-coumarin	BTDEC	350	474	511	34

3-(2-Benzimidazolyl)-7-(diethylamino)-coumarin	BIDEC	333	457	497	28

3-(2-Benzimidazolyl)-7-coumarin	BIC	262	456	497	24

3-(2-Benzimidazolyl)-7-(dipropylamino)-coumarin	BIDPC	361	456	497	24

3-(2-Benzoxazolyl)-7-(diethylamino)-coumarin	BODEC	369	460	502	24

3-(6-methyl-2-benzoxazolyl)-7-(diethylamino)-coumarin	MBODEC	348	458	501	10

3-(Diethylamino)-7-imino-7H-(1)benzopyrano(3',2':3,4) pyrido(1,2-a)benzimidazole-6-carbonitrile	DIBPBC	381	554	580	32

3-(Diethylamino)-7-oxo-7H-(1)benzopyrano(3',2':3,4) pyrido(1,2-a)benzimidazole-6-carbonitrile	DOBPBC	382	559	584	36

The fluorescence properties of coumarin derivatives were measured in DMSO. MW: molecular weight; Ex: fluorescence excitation; Em: fluorescence emission; FI: fluorescence intensity.

### The coumarin derivatives are fixable

**Figure 2:**
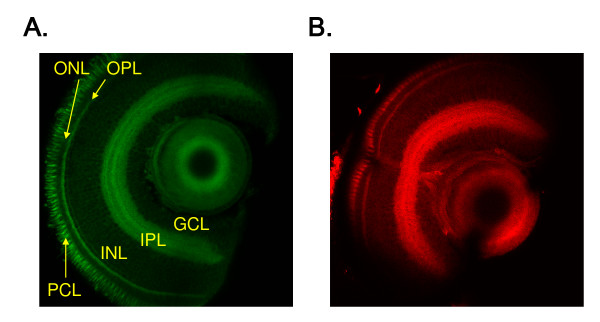
**The coumarin dyes are fixable**. Zebrafish larvae at 6 dpf were stained with BODEC (A) or DIBPBC (B) and fixed in 4% paraformaldehyde. The retinas were sectioned and visualized by confocal laser scanning microscopy. The zebrafish retinas are clearly visualized similar to the *in vivo *imaging.

Many fluorescent small molecules suitable for vital staining are not fixable and thus limit the types of experiments that can be conducted with these molecules [17,24]. To test whether the coumarin derivatives suitable for the *in vivo *imaging could remain within the retina after fixation, zebrafish larvae stained with BODEC or DIBPBC were fixed and sectioned as described in Methods. As shown in Figure 2, both BODEC and DIBPBC still stained the fixed retina and visualized multiple layers, similar to the *in vivo *imaging.

### Staining of rod and UV-sensitive cone photoreceptor cells

**Figure 3:**
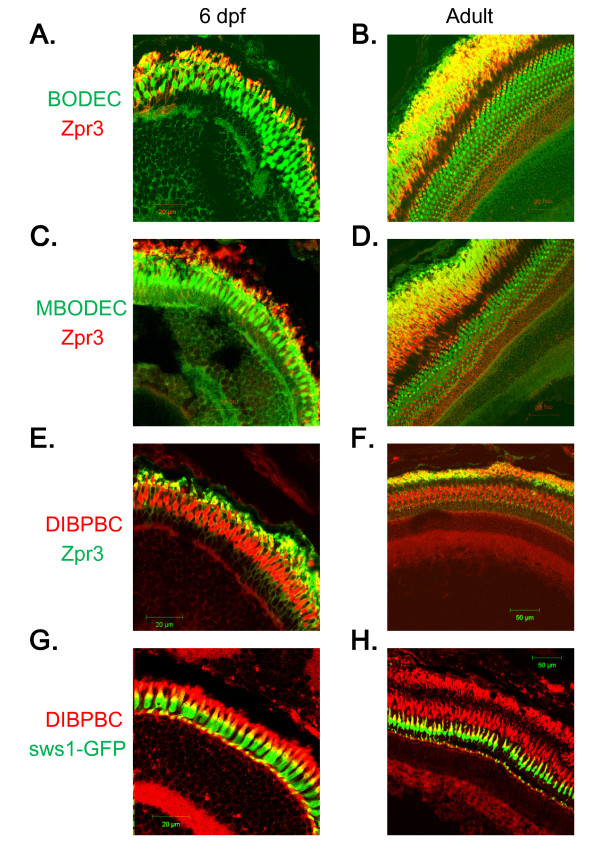
**Staining of rod and UV-sensitive cone photoreceptor cells with the coumarin derivatives**. Retinal sections from wild-type *AB *larvae (A, C and E) and adults (B, D and F) were labeled with zpr3, followed by labeling with secondary antibodies conjugated with Alexa fluorophores and counter-staining with BODEC (A and B), MBODEC (C and D) or DIBPBC (E and F). The fluorescent signals of the coumarin derivatives (green in A-D; red in E and F) partly overlap (yellow in A-F) with those of zpr3 (red in A-D; green in E and F). Retinal sections from a Tg (*sws1*:GFP) larva (G) and adult (H) were counter-stained with DIBPBC. The fluorescent signals of DIBPBC (red) partly overlap (yellow) with those of zpr3 (green).

The zebrafish PCL consists of rods and four classes of cones (i.e. red, green, blue and UV-sensitive) [3]. To delineate the classes of photoreceptors stained with the coumarin derivatives, immunohistochemical analyses were performed as described in Methods. The immunohistochemical analyses revealed that the fluorescent signals of the coumarin derivatives partly co-localized with those of zpr3, a mouse monoclonal antibody that has been widely used to label rod photoreceptor cells in zebrafish [25], suggesting that these coumarin derivatives can at least stain rod photoreceptors (Figure 3A-F).

Next, we utilized a transgenic zebrafish line, Tg (*sws1*:*GFP*), that selectively expresses GFP in UV-sensitive cone photoreceptors under the control of the *sws1 *promoter [14]. *sws1 *encodes the opsin protein, which is selectively expressed in zebrafish UV-sensitive cone photoreceptor cells [26]. Retinal sections from Tg (*sws1*:*GFP*) were counter-stained with DIBPBC whose fluorescence spectrum can be distinguished from that of GFP. As shown in Figure 3G and 3H, the fluorescent signals of DIBPBC partly overlapped with GFP signals of Tg (*sws1*:*GFP*) at both the outer segments and pedicles of UV-sensitive cones, suggesting that DIBPBC can also stain UV-sensitive cone photoreceptors.

### In vivo imaging of retinal development in zebrafish

**Figure 4:**
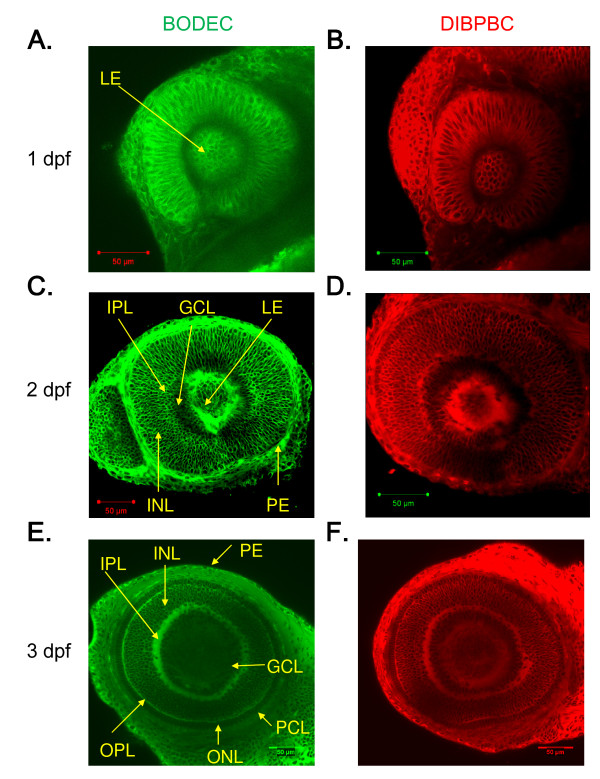
***In vivo *imaging of zebrafish retinal development using the coumarin derivatives**. Zebrafish larvae at 1 dpf (A and B), 2 dpf (C and D) and 3 dpf (E and F) were stained with BODEC (A, C and E) or DIBPBC (B, D and F). After the staining, the retinas were visualized by confocal laser scanning microscopy. The development of the retinal structures is clearly visualized.

To evaluate the ability of the coumarin derivatives for *in vivo *imaging of zebrafish retinal development, embryos at 1, 2, and 3 dpf were immersed in an embryo medium containing 1 μg/ml of BODEC or DIBPBC. At 1 dpf, the lens vesicle detached from the overlying ectoderm (Figure 4A, B). The differentiation of fiber cells was also visualized (Figure 4A, B). At 2 dpf, the IPL could be distinguished from the INL and GCL. The IPL had a reticulated appearance (Figure 4C, D), consistent with a previous reports showing that the IPL includes amacrine cells at this stage [27]. At 3 dpf, the OPL and PCL were also visualized (Figure 4E, F). These findings are consistent with previous studies using *in vitro *approaches such as immunohistochemical analyses [3,27,28].

We also used DIBPBC to stain the transgenic zebrafish lines Tg (*rh*:*GFP*), which selectively expresses GFP in rod photoreceptors under the control of the *rh *promoter [13], and Tg (*huc*:*Kaede*) [29], which selectively expresses the fluorescent protein Kaede in RGC and amacrine cells under the control of the *huc *promoter [30]. The results are shown in Additional file 1: Figure S1 and Additional file 2: Figure S2. At 1 dpf, neither GFP nor Kaede was detected in the retina. At 2 dpf, GFP was detected in a patch of the ventronasal retina and Kaede was detected in RGC inside the IPL. At 3 dpf, the GFP signals increased in the ventronasal patch and Kaede was detected in both RGC and amacrine cells outside of the IPL. At 4 dpf, GFP signals were scattered outside the ventral region. At 5 dpf, the GFP signals outside the ventral region increased further. At all stages, DIBPBC visualized all of the retinas and served as an excellent counter-stain for GFP- and Kaede-expressing retinal cells.

### In vivo imaging of the neuronal disorganization of the zebrafish retina in a genetic model of retinopathy

To examine the feasibility of using coumarin derivatives to visualize the morphological abnormalities in retinal diseases, we used a known genetic zebrafish model of retinopathy, crb2a morphant. *crb2a*, which encodes crumbs homolog 2 protein, is the causative gene of the *oko meduzy *mutant [31]. The *oko meduzy *mutation or knockdown of crb2a causes striking disorganization of the zebrafish retina [31].

**Figure 5:**
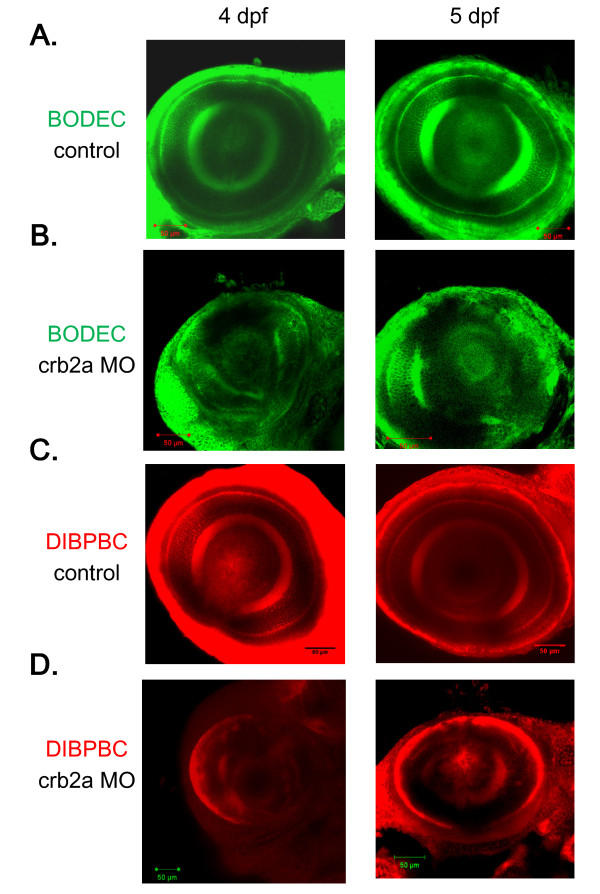
***In vivo *imaging of the zebrafish retina in a genetic model of retinopathy using the coumarin derivatives**. Control zebrafish (A and C) and crb2a morphants (B and D) at 4 and 5 dpf were stained with BODEC (A and B) or DIBPBC (C and D). The retinas were visualized by confocal laser scanning microscopy. The retinal disorganization in the crb2a morphants is clearly visualized by both BODEC and DIBPBC.

We injected an antisense morpholino to knockdown the expression of crb2a [31] in zebrafish embryos. *In vivo *imaging of the crb2a morphants revealed that the layered arrangement of the retinal neurons was drastically disorganized (Figure 5B, D), consistent with a previous study using immunohistochemical analyses [31]. Age-matched controls with no abnormal morphology were evaluated for comparison (Figure 5A, C).

### In vivo imaging of the neuronal disorganization of the zebrafish retina induced by chemical intervention

**Figure 6:**
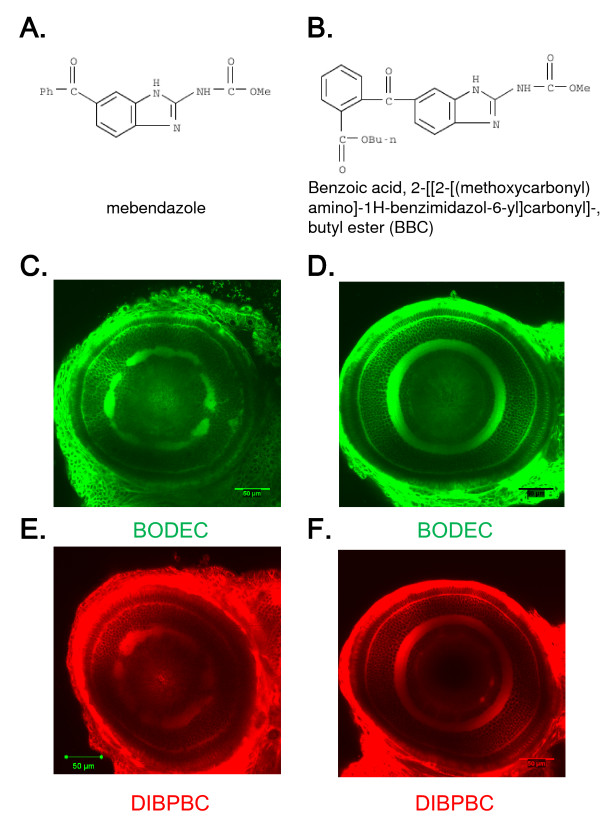
***In vivo *imaging of the zebrafish retina treated with toxic chemicals**. Zebrafish larvae were treated with mebendazole (A) or BBC (B) from 1 to 6 dpf. The larvae were stained with BODEC (C and D) or DIBPBC (E and F). The retinas were visualized by confocal laser scanning microscopy. Both BODEC and DIBPBC clearly visualize the disorganization of the IPL in the zebrafish treated with mebendazole (C and E) but not with BBC (D and F).

It has been reported that numerous clinical drugs cause adverse ocular events by affecting the function and morphology of the retina [32]. The zebrafish has emerged as a versatile animal model to identify potential safety liabilities including visual safety assessments [4]. To examine the feasibility of the coumarin derivatives for detecting drug-induced retinopathy, we treated zebrafish larvae with mebendazole (Figure 6A). Mebendazole is a broad-spectrum anthelmintic compound that adversely affects the organization of the retinal layers in zebrafish larvae [5]. We also used benzoic acid, 2-[[2-[(methoxycarbonyl) amino]-1H-benzimidazol-6-yl]carbonyl]-butyl ester (BBC) (Figure 6B). Although BBC is structurally related to mebendazole, the adverse effects of BBC on the zebrafish retina are much weaker than those of mebendazole [5]. The zebrafish larvae were continuously treated with mebendazole or BBC (both at 0.3 μM) from 1 to 6 dpf. The coumarin derivatives clearly visualized the disorganization of the IPL in larvae treated with mebendazole (Figure 6C, E). The retinal disorganization was severe when zebrafish larvae were treated with 1 μM mebendazole whereas the larvae treated with 0.1 μM mebendazole showed very weak abnormalities (data not shown). No disorganization was observed in larvae treated with BBC (Figure 6D, F).

## Discussion

### Structural characterization of the coumarin derivatives suitable for in vivo imaging of the zebrafish retina

In this study, we were able to distinguish among coumarin derivatives based on their abilities to stain the zebrafish retina. The fluorescence intensities of the coumarin derivatives measured in DMSO were similar (Table 1), suggesting that the differences in the staining abilities may reflect differences in their interactions with target biomolecules in the zebrafish retina and/or their affinities for transporters located at the BRB. We demonstrated that BIDEC, BIC and BIDPC possessing a benzimidazole functional moiety showed comparatively weak staining abilities. According to a previous study [33], the carbonyl group in the coumarin moiety can form complexes with other molecules. The oxygen atom of the carbonyl group in the coumarin moiety and either the sulfur atom in benzothiazole (BTDEC) or the oxygen atom in benzoxazole (BODEC and MBODEC) seem to coordinate with biomolecules by forming a stable intermolecular six-membered ring state. On the other hand, for BIDEC, BIC and BIDPC, the oxygen atom of the carbonyl group in the coumarin moiety and the hydrogen-bonded nitrogen atom in benzimidazole seem to form a stable intra-molecular six-membered ring state. The differences in the interactions with target biomolecules in the zebrafish retina may cause the differences in the staining abilities of the coumarin derivatives. The structural differences among the coumarin derivatives may also result in different permeabilities through the BRB. Previous studies have shown that the permeability of fluorescein through the BRB remains low because active efflux transporters pump out the molecules [34,35]. If the coumarin derivatives with a benzimidazole moiety (i.e. BIDEC, BIC and BIDPC) tend to be ligands for the efflux transporters in the BRB, their staining abilities of the retina may be low. Further studies are required to elucidate the structure-staining relationships.

### Comparison of fluorescent small molecules for vital staining of the zebrafish retina

Bodipy-ceramide and inorganic nanocrystal fluorophores such as quantum dots (QD) have been used for *in vivo *imaging of the zebrafish retina [16,17]. The coumarin derivatives identified in this study differ from these fluorescent molecules in several aspects. First, the coumarin derivatives preferentially distribute to neuronal tissues including retinal cells and brain in zebrafish larvae, suggesting that the coumarin derivatives can penetrate both BRB and BBB. The mechanism is currently unknown. Bodipy-ceramide not only stains the plasma membranes of all cells in zebrafish larvae but also localizes to the interstitial fluid [16]. Although the intracellular distribution of QD in zebrafish larvae is dependent on the QD coating [17], we could not find any reports showing selective tissue distribution of QD. Therefore, the preferential neuronal distribution of coumarin derivatives is a significant feature. Second, the vital staining using the coumarin derivatives is fast and cheap. The *in vivo *imaging using coumarin derivatives requires 30 min of staining at 1 μg/ml (about 3 μM), whereas the *in vivo *imaging using Bodipy-ceramide requires 2-8 hours of staining at 50-100 μM [19,20]. These differences suggest that the fluorescence intensities of the coumarin derivatives in the retina may be stronger than that of Bodipy-ceramide owing to preferential accumulation of the coumarin derivatives in the neuronal tissues. Third, the fluorescence spectra of the coumarin derivatives are well separated from those of GFP and red fluorescent protein, making it possible to counter-stain transgenic zebrafish. The fluorescence spectrum of QD can be controlled by the constituent materials, particle size and surface chemistry [36], making QD suitable for counter-staining of transgenic zebrafish expressing fluorescent proteins [17]. However, QD cannot be absorbed into zebrafish larvae and require intra-retinal injection of the QD. These features render the coumarin derivatives well-balanced labeling agents for *in vivo *imaging of the zebrafish retina and powerful tools for genetic and chemical screenings using the zebrafish retina.

### Potential of the coumarin derivatives for in vivo imaging of mammalian retinas

Although rodents are nocturnal animals with poor color vision, they have been standard laboratory models for retinal research [2]. The current methods for *in vivo *imaging of rodent retinas often require either retrograde or intravitreal injection of fluorescent probes such as cyanine dyes to visualize RGC [37]. These injections are invasive and may require the administration of probes to be repeated for long-term assessment [37]. It would be very valuable if the less invasive administration of the coumarin derivatives identified in this study would also be able to stain rodent retinas. The preliminary experiments using mouse suggest that the coumarin derivatives intravenously injected can penetrate the BRB and stain multiple layers of the mouse retina (data not shown). Further studies are required to examine whether the coumarin derivatives can be used for *in vivo *imaging of the rodent retina.

## Conclusions

In this study, we have identified coumarin derivatives that can preferentially stain retinal cells, thereby enabling visualization of the multiple layers in the retina in living zebrafish. These coumarin derivatives can be applied to genetic and chemical screenings using zebrafish to identify genes and chemicals that may affect retinal development and the pathogenesis of retinal diseases. The structural characterization of the coumarin derivatives may also provide useful information to identify chemicals that can accumulate in retinal cells.

## Methods

### Zebrafish strains

Zebrafish were bred and maintained according to the methods described by Westerfield [38]. Briefly, zebrafish were raised on 14-h/10-h light/dark cycle at 28.5 ± 0.5°C. Embryos were obtained via natural mating and cultured in egg water. All experiments in this study were carried out according to the ethical guidelines established by the Institutional Animal Care and Use Committee at Mie University. Embryos older than 24 hours post-fertilization (hpf) were treated with 200 μM 1-phenyl-2-thiourea (PTU) to block pigmentation. Transgenic zebrafish lines expressing GFP under the control of the *sws1 *and *rh *promoters and Kaede under the control of the *huc *promoter were obtained from the National BioResource Project for Zebrafish (http://www.shigen.nig.ac.jp/zebra/index_en.html).

### Compounds

All the fluorescent coumarin derivatives (BODEC, MBODEC, BTDEC, DIBPBC, BIDEC, BIC, BIDPC and DOBPBC) examined were obtained from Canon Inc. (Tokyo, Japan). Stock solutions of the coumarin derivatives were prepared by dissolution in DMSO at 1 mg/ml. Mebendazole was purchased from Sigma-Aldrich (St. Louis, MO). BBC was obtained from Namiki Shoji Co. Ltd. (Tokyo, Japan).

### In vivo imaging of the zebrafish retina

Zebrafish larvae were exposed by immersion in egg water containing 1 μg/ml of a coumarin derivative for 30 min at 28.5 ± 0.5°C. After a brief wash with egg water, the larvae were anesthetized with 0.016% tricaine methanesulfonate (MS-222) and transferred onto glass slides. A few drops of 2% low-melting agarose containing 0.016% MS-222 were laid over the larvae and the larvae were immediately oriented on the lateral side. The retinas of the embedded larvae were observed using a Zeiss 510 confocal laser scanning microscope. Images were captured at a resolution of 512×512 pixels using a 20× (NA 0.75) or 40× (NA 1.2) water immersion objective lens. The images were processed with Image J (http://rsbweb.nih.gov/ij/index.html).

### Immunohistochemical analysis

Zebrafish were fixed in Histo-Fresh (Falma, Tokyo, Japan) at 4°C overnight. The larvae were then immersed in 30% sucrose solution at room temperature (RT) for 5 hours, and mounted in 100% Tissue-Tek OCT (Sakura Finetechnical, Tokyo, Japan) for 1 hour at RT. The retinas of adult zebrafish were mounted in 100% Tissue-Mount (Sakura Finetechnical) at 4°C overnight. These zebrafish were frozen using liquid nitrogen and isopentane and sectioned using a microtome (Leica, Tokyo, Japan). Sections of 8 μm in thickness were adhered to MAS-coated glass slides (Matsunami Glass, Osaka, Japan), dried at RT for 30 min and stored at -80°C until use. For immunofluorescence labeling, the sections were incubated at RT for 30 min, dried and washed with phosphate-buffered saline (PBS). The sections were then blocked for 1 h in a blocking solution (Nakalai Tesque, Kyoto, Japan). The blocking solution was replaced with a primary antibody diluted with Can Get Signal Solution A (Toyobo, Osaka, Japan) and incubated overnight at 4°C. The sections were washed three times with PBS containing 0.2% Triton X-100 (PBS-T), followed by incubation for 1 h at RT with fluorescent secondary antibodies (Invitrogen) diluted with Can Get Signal Solution A. A coumarin compound (1 μg/ml of BODEC, DIBPBC or MBODEC) was also included in the secondary antibody solution. The sections were washed three times with PBS-T, and mounted with Fluoromount G (Southern Biotech, Birmingham, AL). The immunolocalized antigens were visualized using a Zeiss 510 confocal laser scanning microscope. The following antibodies were used: zpr3 (1:10; Zebrafish International Resource Center, Eugene, OR); anti-mouse IgG conjugated with Alexa 488 for co-staining with BODEC and MBODEC or Alexa 543 for co-staining with DIBPBC (1:100 each; Invitrogen, Carlsbad, CA).

### Microinjection

Zebrafish embryos at the one-to-four cell stage were microinjected with an antisense morpholino for *crb2a *[31]. The embryos were raised until 4 and 5 dpf in embryo water containing 200 μM PTU and subjected to the *in vivo *imaging.

## Authors' contributions

KW and YN participated in the study design, carried out the experiments and wrote the manuscript. TO, TK, MH and YS participated in the study design and carried out the experiments. TN and TS participated in the study design and helped to draft the manuscript. NU, JK, ZW and ZZ helped to carry out the experiments. NN, TM and TI conceived the study. TT conceived the study and wrote the manuscript. All the authors have read and approved the final version of the manuscript.

## Supplementary Material

Additional file 1 **Figure S1: *In vivo *imaging of the zebrafish retina by combining the coumarin derivatives and transgenic zebrafish expressing GFP in rod photoreceptor cells**. Tg (*rh*:*GFP*) zebrafish from 1 to 5 dpf were stained with DIBPBC. The retinas were visualized by confocal laser scanning microscopy. The development of rod photoreceptor cells is visualized with high resolution by the counter-staining with DIBPBC. Click here for file

Additional file 2 **Figure S2: *In vivo *imaging of the zebrafish retina by combining the coumarin derivatives and transgenic zebrafish expressing Kaede in retinal ganglion cells and amacrine cells**. Tg (*huc*:*Kaede*) zebrafish from 1 to 5 dpf were stained with DIBPBC. The retinas were visualized by confocal laser scanning microscopy. The development of retinal ganglion cells and amacrine cells is visualized with high resolution by the counter-staining with DIBPBC. Click here for file
