# Supplementary figures and images for: In vivo imaging of zebrafish retinal cells using fluorescent coumarin derivatives
Source: BMC Neurosci. 2010 Sep 15;11:116. doi: 10.1186/1471-2202-11-116 (PMC2945357; doi:10.1186/1471-2202-11-116)

## Slide 1
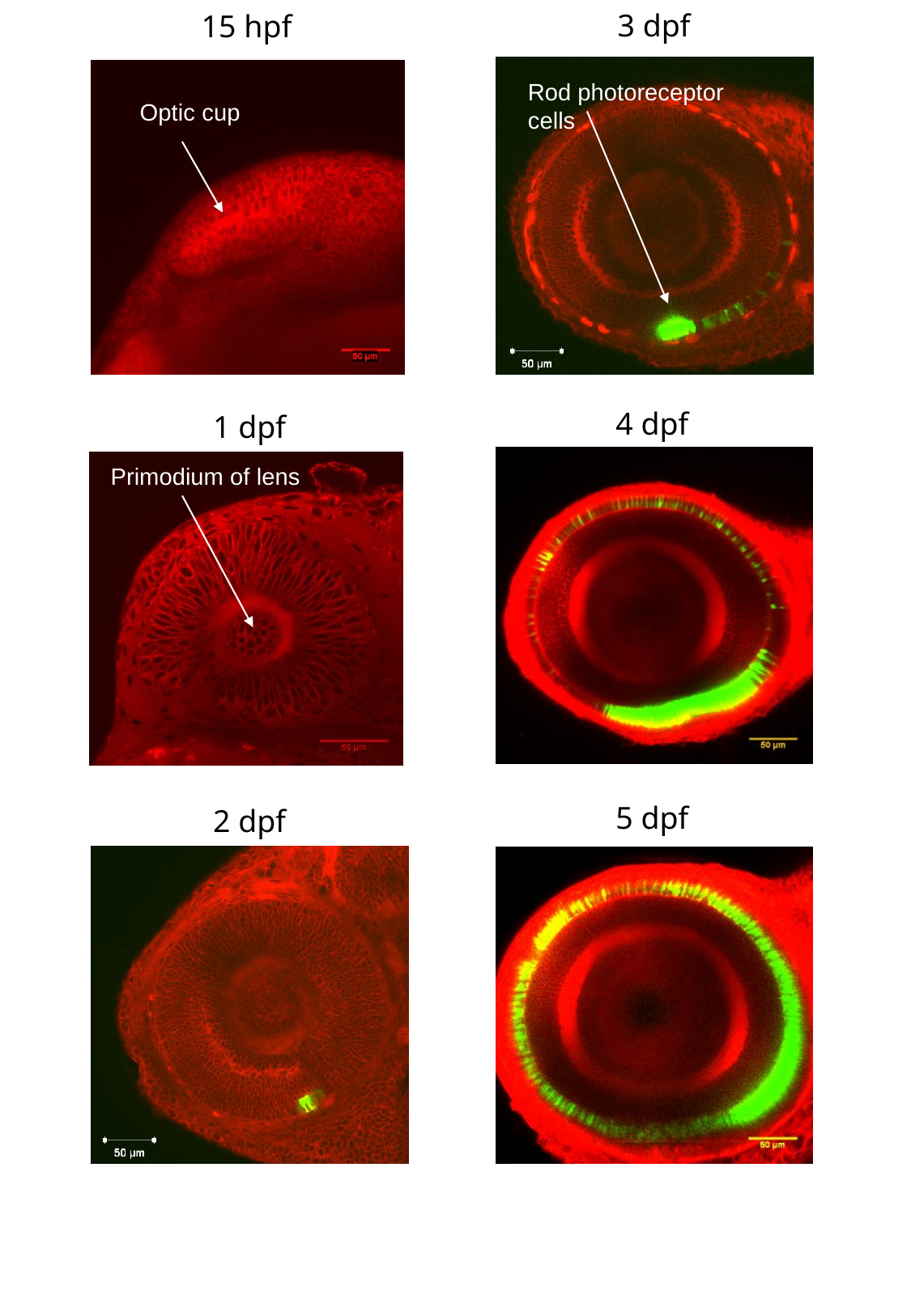

3 dpf
15 hpf
Rod photoreceptor
cells
Optic cup
4 dpf
1 dpf
Primodium of lens
5 dpf
2 dpf

Supplement: Additional file 1 — Figure S1: In vivo imaging of the zebrafish retina by combining the coumarin derivatives and transgenic zebrafish expressing GFP in rod photoreceptor cells. Tg (rh:GFP) zebrafish from 1 to 5 dpf were stained with DIBPBC. The retinas were visualized by confocal laser scanning microscopy. The development of rod photoreceptor cells is visualized with high resolution by the counter-staining with DIBPBC. [file 1471-2202-11-116-S1.PPT]
