# Supplementary material for: In vivo imaging of zebrafish retinal cells using fluorescent coumarin derivatives
Source: BMC Neurosci. 2010 Sep 15;11:116. doi: 10.1186/1471-2202-11-116 (PMC2945357; doi:10.1186/1471-2202-11-116)
Supplement: Additional file 2 — Figure S2: In vivo imaging of the zebrafish retina by combining the coumarin derivatives and transgenic zebrafish expressing Kaede in retinal ganglion cells and amacrine cells. Tg (huc:Kaede) zebrafish from 1 to 5 dpf were stained with DIBPBC. The retinas were visualized by confocal laser scanning microscopy. The development of retinal ganglion cells and amacrine cells is visualized with high resolution by the counter-staining with DIBPBC. [file 1471-2202-11-116-S2.PPT]

## Slide 1
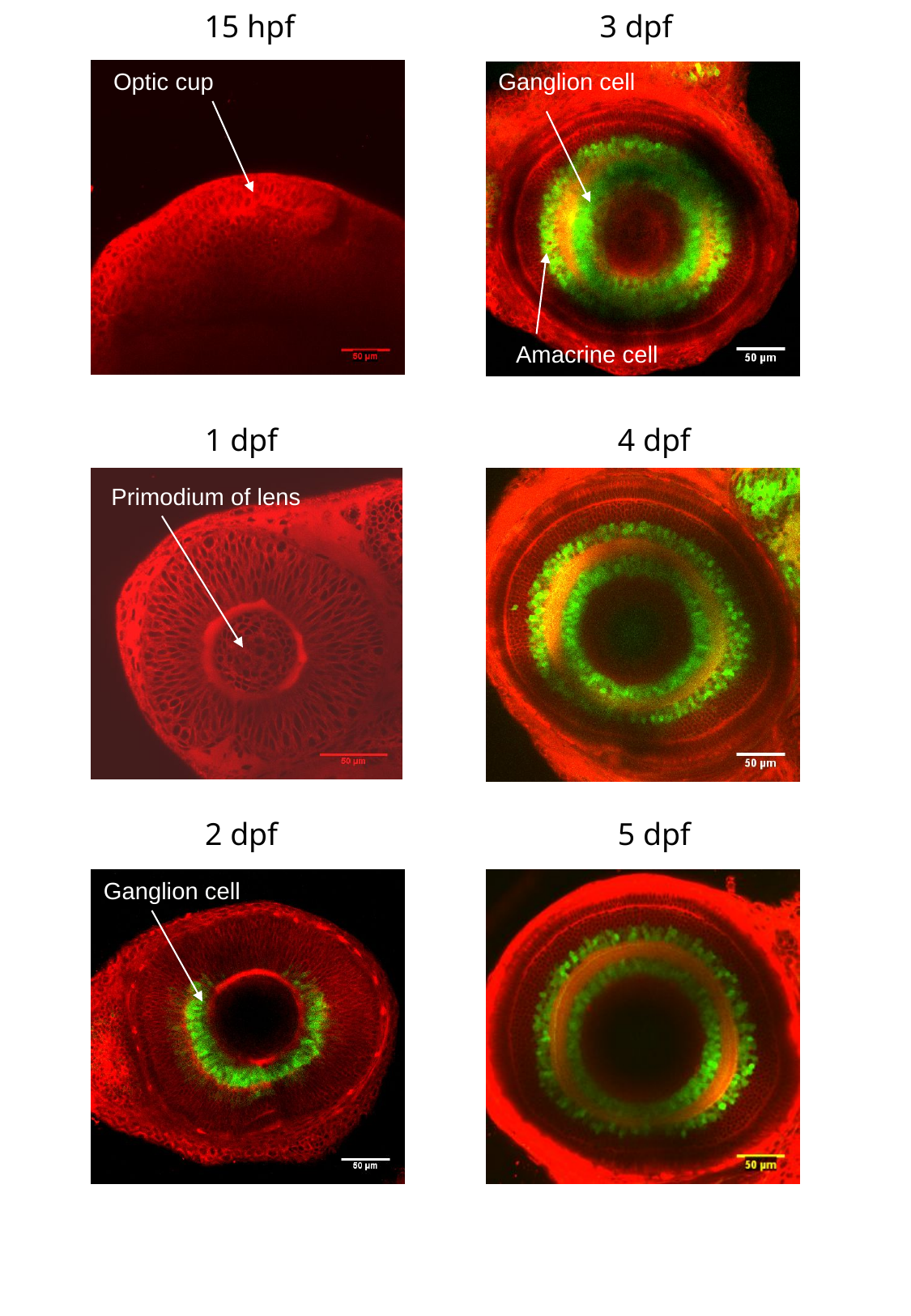

15 hpf
3 dpf
Optic cup
Ganglion cell
Amacrine cell
1 dpf
4 dpf
Primodium of lens
2 dpf
5 dpf
Ganglion cell
